# Insulation Failure Quantification Based on the Energy of Digital Images Using Low-Cost Imaging Sensors

**DOI:** 10.3390/s20247219

**Published:** 2020-12-16

**Authors:** Jordi-Roger Riba, Álvaro Gómez-Pau, Manuel Moreno-Eguilaz

**Affiliations:** 1 Electrical Engineering Department, Universitat Politècnica de Catalunya, 08222 Terrassa, Spain; 2 Electronics Engineering Department, Universitat Politècnica de Catalunya, 08222 Terrassa, Spain; alvaro.gomez-pau@upc.edu (Á.G.-P.); manuel.moreno.eguilaz@upc.edu (M.M.-E.)

**Keywords:** imaging sensor, digital images, image processing, energy, partial discharges, corona effect, high voltage, low pressure

## Abstract

Insulation faults in high-voltage applications often generate partial discharges (PDs) accompanied by corona activity, optical radiation mainly in the ultraviolet (UV) and visible bands. Recent developments in low-cost, small-size, and high-resolution visible imaging sensors, which are also partially sensitive to the UV spectral region, are gaining attention due to their many industrial applications. This paper proposes a method for early PD detection by using digital imaging sensors, which allows the severity of insulation faults to be assessed. The electrical power dissipated by the PDs is correlated to the energy of the acquired visible images, and thus, the severity of insulation faults is determined from the energy of the corona effect. A criterion to quantify the severity of insulation faults based on the energy of the corona images is proposed. To this end, the point-to-plane gap configuration is analyzed in a low-pressure chamber, where digital image photographs of the PDs are taken and evaluated under different pressure conditions ranging from 10 to 100 kPa, which cover the typical pressure range of aeronautic applications. The use of digital imaging sensors also allows an early detection, location and quantification of the PD activity, and thus assessing the severity of insulation faults to perform predictive maintenance tasks, while enabling the cost and complexity of the instrumentation to be reduced. Although the approach proposed in this paper has been applied to detect PDs in aeronautic applications, it can be applied to many other high-voltage applications susceptible of PD occurrence.

## 1. Introduction

Insulation materials typically used in electric wires suffer different fault modes, including arc tracking, arcing and insulation flashover in increasing order of severity [1], which generate PDs and corona effects [2]. Whereas PDs are a type of electrical discharges which do not totally bridge the insulation separating two conductive electrodes [3], corona discharges are a specific type of partial discharges due to the ionization of a gas (insulation medium) surrounding a surface of high electric potential. Arcing and arc tracking in small-size gaps are typically preceded by corona activity generated in the area where the electric field strength is maximum [3,4,5]. Surface discharges, which are often responsible of failures in electronic systems intended for low-pressure applications [6] also generate corona discharges. Therefore, corona discharges produce premature faults in insulation materials, especially when operating at low pressure [7], because air density plays a key role in the development of electrical discharges [8].

The next generations of more electric aircrafts (MEA) will require the weight to be reduced [9], lowering operation and maintenance costs, system complexity, greenhouse gas emissions and fuel consumption with respect to current aircrafts [10]. To achieve these goals, MEAs will require more electrical power than current aircrafts [11], thus requiring operation at higher voltages [12], which will be above 1 kV [7], higher power densities and reduced wire distances. The dielectric strength reduces with air pressure, thus promoting ionization processes such as PDs, corona, arcing and arc tracking. Due to the low operating pressures, electrical systems appropriately designed for ground operations can be exposed to important levels of PDs in aircraft environments [13]. Low-pressure environments favor premature insulation ageing and degradation [14]. These particularities promote new failure modes, such as a higher probability of electric arc appearance, with the associated harmful effects [15]. PDs produce transformations in insulation systems which generate a conductive path across the insulation, with the consequent temperature rise due to the flow of an electric current, weakening the insulation and facilitating the formation of an electrical arc or even a complete breakdown [16]. The solid insulation layer of electrical wires can be carbonized due to the electric current associated with the arcing phenomenon generated between two wires, which is known as arc tracking. Protections against arc tracking for aeronautic installations are not totally effective due to the low level of activity in the early fault stages. Due to its low level, the fault current generated by PDs and corona usually remain undetected by existing protections, although their continuous effect damage insulation systems until complete failure is produced [17]. Electrical systems for MEA aircrafts offer increased power density, although they must comply with the safety and reliability requirements. In this context, arcing and arc tracking are among the major issues to be solved [18]. However, the impact of arc tracking on future MEAs is still not well understood [19]; therefore, it deserves exhaustive research plans.

Insulation faults are not often directly evaluated. Instead, some measurable physical effects can be analyzed [20], such as acoustic noise, electromagnetic radiation, radio interference voltage, ultraviolet or visible emissions, generation of chemical components such as ozone or others or temperature changes. There is an imperative need to evaluate the severity of insulation faults in existing installations, since it is vital for ensuring a safe, reliable and stable operation of power systems. It is well known that PDs often generate optical radiation [20] in the ultraviolet (UV), visible and infrared bands [21,22], whereas the spectral content of the discharges depends upon the type of electrode and the nature of the discharges. Many research works focusing on the analysis of optical emissions generated by PDs analyze the UV band [23,24]. However, the use of UV cameras is still problematic, due to their high cost, issues related to noise performance or limited image resolution, thus leading to unsatisfactory spatial resolution and PD source location accuracy [20]. Although hybrid UV-visible cameras based on the latest fusion technology merging both visible and UV images are commercially available, its industrial application becomes very difficult because of their high volume and weight and prohibitive cost. The visible light emitted by PDs can be acquired by means of digital imaging sensors or digital cameras [25]. This strategy is appealing since many industrial applications already use visible cameras, whereas the use of UV cameras is much less widespread. In addition, industrial applications are increasingly applying visible cameras due to the fast evolution of visible imaging sensors [26]. With regard to the aforementioned and the fact that visible images of PDs contain rich information, it is highly appealing to study and characterize the PDs by analyzing the information contained in visible images of the PDs [20]. Nowadays, this strategy can be implemented due to the latest developments of small-size, low-cost and high-resolution visible imaging sensors, most of which are also partially sensitive to specific regions of the UV spectrum [4].

Electronic arc fault circuit breakers (AFCBs) are being used by existing commercial aircrafts and in household installations to protect cabling systems against arcing effects. Similar devices known as arc fault circuit interrupter (AFCI) or arc fault detector (AFD) are used to protect domestic and industrial electrical installations against the harmful effects of electric arcs to minimize fires occurrence. Several factors, including inverter noise and switching harmonics, antenna effect, crosstalk or system topology among others, impact the arc signal, thus producing false trips on AFDs and AFCIs [27]. AFCBs analyze the current waveform, which is altered due to the electrical arcing activity, identify the fault condition and, they must trip within 100 ms [28]. The main problem is that current AFCBs react after arcing occurrence, that is, when the insulation has suffered some level of damage, so they are not able to anticipate the fault condition. In addition, commercial industrial protections currently applied for detecting and protecting against arcing activity are not able to totally remove the associated transients [29] and cannot determine the exact location of the fault. Therefore, there is still much room to improve current AFCBs in order to locate the fault and to anticipate arcing activity and thus to minimize the associated degradation in insulation systems and the related risks. Since next generations of MEA aircrafts are expected to make a more intense use of electronic and electrical systems, AFCBs use is problematic [30], thus requiring new developments to ensure an early detection of very incipient faults. The approach proposed in this paper tries to fill this gap of knowledge and technical developments. We present a simple system based on calculating the energy of the optical emissions related to corona, which is a pre-arc phenomenon, to identify and detect pre-arcing faults in the very early stage, much before arcing phenomenology is developed, thus preventing cabling systems and electric circuits of serious damaging.

Modern widebody aircrafts include hundreds of thousands of electrical wiring interconnecting system (EWIS) components, comprising connectors, splices, clamps, terminations and other wiring support equipment and hundreds of kilometers of wires and cables. Therefore, to ensure a safe and reliable operation the frequency of component inspections should be increased along with the best analysis and assessment capabilities. The aerospace industry is very active in developing health monitoring systems, but problems in wiring systems often remain hidden. When a component reports an error, the first maintenance operation consists of replacing the failed component, but often, after inspecting and testing the component, no fault is found, which is known as No-Fault Found. The cycle can repeat for many iterations before maintainers find the origin of the problem. Some of these incidents can lead in the grounding of the aircraft, aborted takeoffs or emergency landing [31].

Methods for arc fault detection are mostly based on the study of the particular shape of the electric current waveform, known as arc signature. To this end, diverse identification strategies have been proposed, including the use of neural networks, Kalman filters, fuzzy logic [32], wavelet decomposition or discrete Fourier transform [33] among others. However, such methods need a preliminary learning stage, with the inherent associated difficulties due to the large number of experiments required, which could not be exhaustive [32].

By determining the intensity of the discharges, the severity of insulation faults can be indirectly inferred. In this line, the primary purpose of this paper is to develop an automated method to assess the severity of insulation faults by analyzing the visible UV light emitted by the PDs. To this end, this paper determines the energy content of digital images of the visible UV corona emissions generated by the PDs, which is correlated to the electrical power dissipated by the discharges, which are acquired using a low-cost high-resolution imaging sensor. This is a new approach with respect the state of the art. advantages of the proposed method include immunity to switching harmonics, lightning strikes and electromagnetic noise, thus enabling the number false positives (nuisance tripping) to be lowered, since commercial protections in some circumstances identify regular circuit behaviors as arc activity. In addition, the proposed strategy also allows the source of PDs to be identified and located much before the damaging arcing occurrence, thus anticipating the fault and offering the possibility to complement existing commercial protections such as AFCBs, which only act when the arc has developed. Although the approach proposed in this paper has been tested in a simulated aircraft environment, it can be applied to many other industrial applications.

The rest of the article is organized as follows. Section 2 is devoted to present the details of digital image processing in order to compute their associated energy. The used experimental setup, allowing the high-voltage measurements under different pressure conditions, is detailed in Section 3. Section 4 presents the obtained results as well as their discussion. Finally, Section 5 summarizes and concludes the paper.

## 2. Applied Digital Image Processing Techniques

A digital image, *I_m_*_×*n*_, of dimensions *m*-by-*n* includes *m* pixels in the vertical axis and *n* pixels in the horizontal axis; *I*(*i,j*) designates the *i*-th and *j*-th horizontal and vertical image coordinates, respectively. Therefore, the image includes a total of *N* = *m*·*n* pixels, where 1 ≤ *i* ≤ *m*, 1 ≤ *j* ≤ *n* [34].

RGB images are stored as *m*-by-*n*-by-3 data arrays, defining the red, green, and blue color components of all individual pixels.

(1) $$I_{RGB}(i,j,l)\;=\;\left\{\right.\underset{R(i,j)}{\underbrace{I_{RGB}(i,j,1)\;}},\;\underset{G(i,j)}{\underbrace{I_{RGB}(i,j,2)}},\;\underset{B(i,j)}{\underbrace{I_{RGB}(i,j,3)}}\left.\right\}$$

where *i* = 1,2,…,*m*, *j* = 1,2,…,*n* and *l* = 1,2,3.

**Figure 1:**
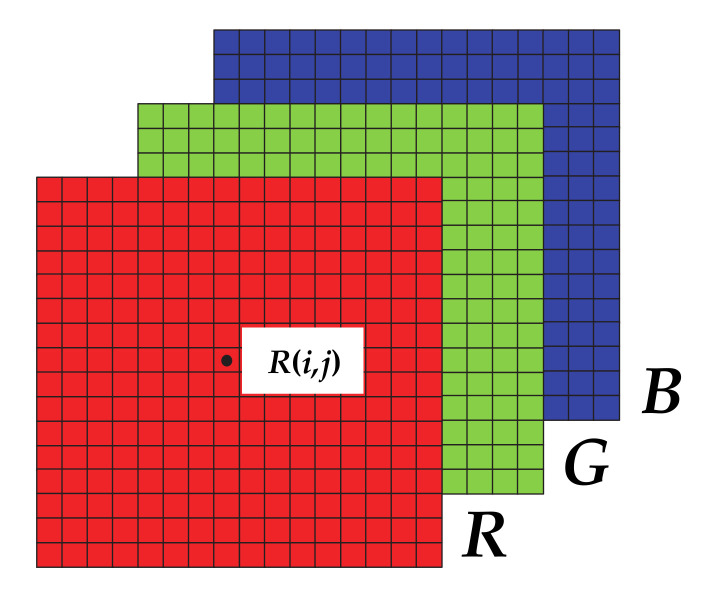
Data structure of RGB images.

Figure 1 shows the data structure of a RGB image showing each of the 3 image color slices or channels.

The method proposed in this paper computes the energy of the images based on the gray-level intensity of all pixels in the image, and thus, to determine the energy of the image, the RGB image needs to be converted to a grayscale image, by applying the following transformation,

(2) $$I_{grayscale}(i,j)=0.299\cdot R(i,j)\;+\;0.587\cdot G(i,j)\;+\;0.114\cdot B(i,j)$$

where the values of the three channels *R*(*i,j*), *G*(*i,j*) and *B*(*i,j*) usually fall in the range 0 to 255, corresponding to 8-bit unsigned integers. The weights of the three channels (*R,G,B*) in (2) are according to the human luminous efficacy function, which depends on the spectral sensitivity of a typical human eye. It is worth noting that the values of the three channels *R*(*i,j*), *G*(*i,j*) and *B*(*i,j*) can be affected by diverse factors, such as the intensity of the background light, exposure time of electromagnetic noise among others.

The energy of the image can be defined as,

(3) $$E(I_{grayscale})=\sum_{i=1}^{m}\sum_{j=1}^{n}I_{{}_{grayscale}}^{k}(i,j)$$

where *k* is an exponent, its value being either 1 [35] or 2 [36] in most references, *k* = 2 being inherited from the signal processing field [37], although in this paper it is assumed *k* = 1, as demonstrated in Section 4. Although there are different definitions of the energy of an image, we have selected the one shown in (3) with *k* = 1. It assumes that the value of each pixel of the grayscale image is proportional to the power density (W/m^2^) of the incident light integrated over the pixel area (m^2^) and exposure time (s), thus resulting in units of energy (J). Therefore, the energy of the image can be easily calculated by adding the grayscale value of all pixels in the image.

In order to compare the energy content of different images and to avoid large values of the energy, it is proposed to normalize the energy as follows,

(4) $$E_{normalized}(I_{grayscale})=\frac{\sum_{i=1}^{m}\sum_{j=1}^{n}I_{{}_{grayscale}}^{}(i,j)}{n\cdot m\cdot 255}\cdot 100$$

**Figure 2:**
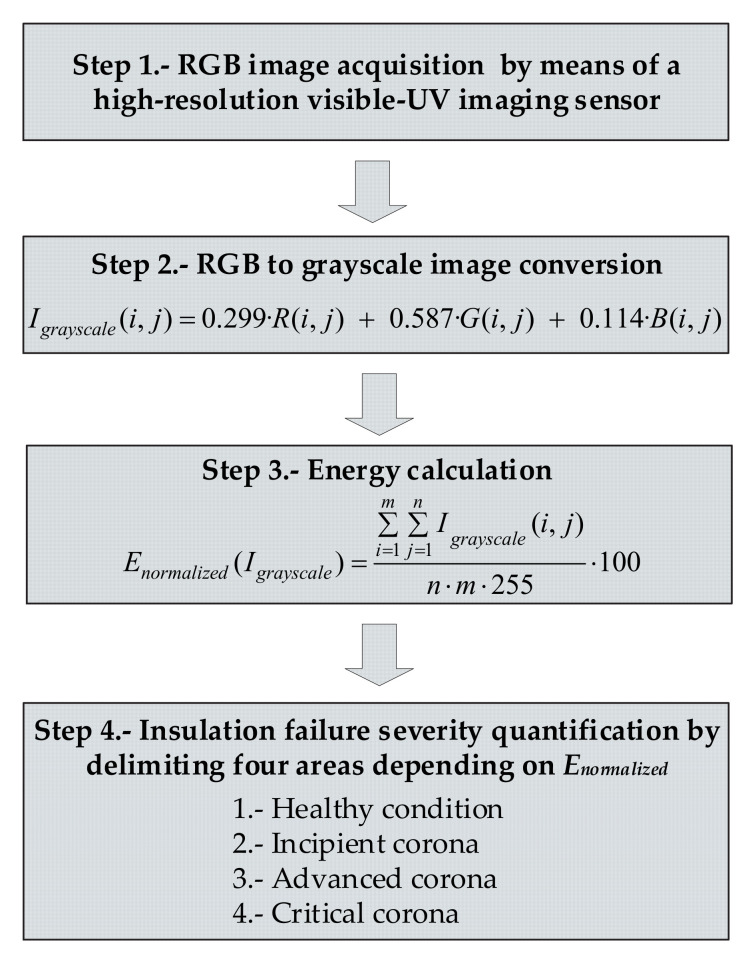
Process diagram to determine the severity of the insulation failure.

The following lines describe the simple Matlab^®^ code proposed to determine the energy of a RGB image once it has been converted to grayscale,

R = image_RGB (:,:,1);
G = image_RGB (:,:,2);
B = image_RGB (:,:,3);
m = size(Image_RGB,1);
n = size(Image_RGB,2);
image_GRAY = 0.299 * R + 0.587 * G + 0.114 * B;
energy = sum(sum(image_GRAY));
energy_normalized = 100*energy/(n*m*255);

In this paper the energy content of the digital corona images is determined by applying the statistical image analysis approach summarized in Figure 2.

The approach proposed in this work and summarized in Figure 2 consists of the following steps,

Step 1. Image acquisition using a high-resolution image sensor. The long-exposure photographs were taken for 32 s long using ISO 400 sensitivity, with manual focus, automatic white balance and RGB mode.
Step 2. All RGB images are converted to grayscale by applying the transformation in (2).
Step 3. The normalized energy of each image is calculated by applying (3).
Step 4. As it will be proved in Section 3, the energy of an image is proportional to the power dissipated by the partial discharges, thus allowing the severity level of insulation faults to be quantified on four levels, i.e., healthy condition, incipient corona, advanced corona, and critical corona.

## 3. Experimental Setup

PD experiments were conducted in a low-pressure chamber at a constant temperature of 20 °C, and the pressure was changed from 100 kPa (1 atm) to 10 kPa (0.1 atm), to simulate, respectively, the pressure conditions between sea level and an altitude of 16 km, which covers the altitude and pressure range of commercial and military aircrafts.

**Figure 3:**
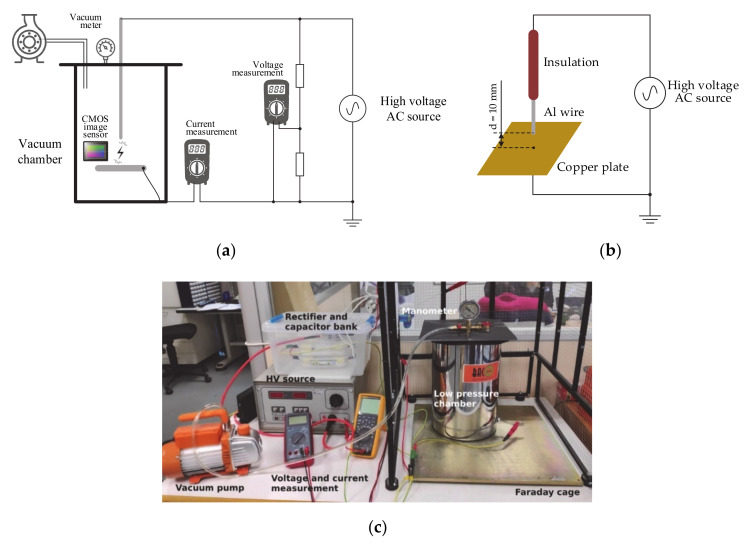
(**a**) Experimental setup. (**b**) Point-to-plane gap. (**c**) Faraday cage with the low-pressure chamber, the vacuum pump, and the instrumentation.

A stainless steel low-pressure cylindrical chamber was used in the experiments. It has 375 mm height and 130 mm diameter, these dimensions allow the electronic and electrical components needed to generate and detect the electrical discharges to be fitted, as well as to transmit wirelessly the long-exposure photographs to an external personal computer. The pressure in the chamber was reduced by means of a single stage vacuum pump (Bacoeng BA-1, Bacoeng, Suzhou, Jiangsu, China, 0.085 m^3^/minute, 1/4 HP). The low-pressure chamber was placed inside a metallic Faraday cage, as shown in Figure 3.

The DC high-voltage was generated by means of an adjustable AC high-voltage source (Tecnolab RD-6, Tecnolab, Barcelona, Spain, 10 kV_RMS_, 600 VA) connected to a reversible diode rectifier and a high-voltage smoothing capacitor. This setup enables a flat positive and negative DC high voltage to be generated. A 1000:1 resistive voltage divider was used to measure the output DC voltage, which was connected to a calibrated digital multimeter in DC voltage mode. The leakage current was measured by means of a calibrated digital multimeter [38] in DC current mode connected in series with the circuit, as shown in Figure 3.

The point-to-plane gap is composed of a vertical 16 AWG tin-plated Al conductor. The tip of the conductor was placed at a height of 10 mm above a grounded flat copper plane. Point-to-plane arrangements are reference gaps in high-voltage applications [39], allowing the generation of PDs.

Figure 3 shows the experimental setup used to acquire the generate PDs and to acquire digital corona images.

This work analyzes the visible UV light emitted by the corona effect, because corona represents an early pre-arc condition. Chemical reactions induced by corona activity tend to produce a gradual damage in wire insulation, thus eventually producing arc tracking and arcing phenomena, whose effects are very damaging and harmful [40]. Digital visible UV corona images were acquired by means of a high-resolution imaging sensor (Sony IMX586 backlit stacked CMOS sensor, Sony, Minato City, Tokyo, Japan, 48 Mpixels, unit cell dimensions 0.80 μm × 0.80 μm, sensor size 8.0 mm diagonal, lens focal 17.9 mm). To detect the corona effect, long-exposure photographs were taken (32 s exposure, ISO 400, manual focus mode, automatic white balance). It is worth noting that backlit CMOS sensors are sensitive to both visible and UV spectra [41].

## 4. Experimental Results and Discussion

The experimental results attained in this work are summarized in this section from the gap geometry and using the instrumentation detailed in Section 3.

### 4.1. Long-Exposure Photographs

**Figure 4:**
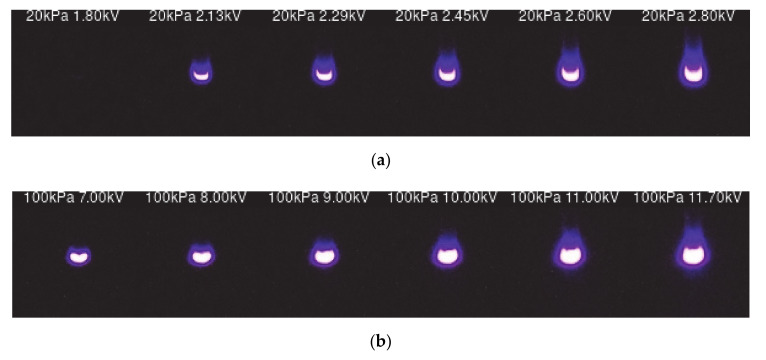
Positive DC corona images taken at different conditions. (**a**) At 20 kPa applying from left to right 1.8 kV, 2.13 kV, 2.29 kV, 2.45 kV, 2.60 kV and 2.80 kV. (**b**) At 100 kPa applying from left to right 7.0 kV, 8.0 kV, 9.0 kV, 10.0 kV, 11.0 kV and 11.7 kV.

**Figure 5:**
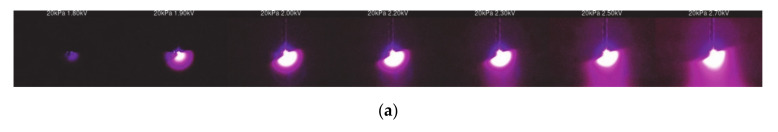

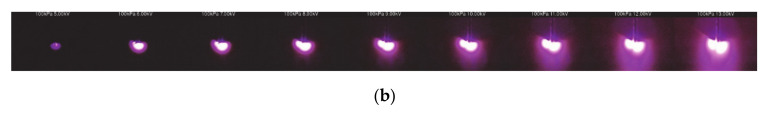
Negative DC corona images taken at different conditions. (**a**) At 20 kPa applying from left to right 1.8 kV, 1.9 kV, 2.0 kV, 2.2 kV, 2.3 kV, 2.5 kV and 2.7 kV. (**b**) At 100 kPa applying from left to right 5.0 kV, 6.0 kV, 7.0 kV, 8.0 kV, 9.0 kV, 10.0 kV, 11.0 kV, 12.0 kV and 13.0 kV.

Long-exposure photographs (32 s long, RGB mode, ISO 400, manual focus, automatic white balance) of the setup detailed in Section 3 were taken to prove the hypothesis made in this paper under stabilized positive and negative DC voltages. To this end, photographs were taken in the range 10–100 kPa in increments of 10 kPa, for both positive and negative DC supply, some of which are shown in Figure 4 and Figure 5. Several images at different voltage levels were acquired for each pressure level. They accounted for the evolution of the intensity of the corona effect, from very low intensity to very high intensity corona effect. Figure 4 and Figure 5 show some of the acquired images for both positive DC and negative DC corona, respectively, which were acquired using the low-pressure chamber and the setup detailed in Section 3.

### 4.2. Electrical Measurements: Leakage Current versus Applied Voltage

The electrical power dissipated by the PDs (*P_PD_*) generated under DC supply can be determined from the product of leakage current (*I_leakage_*) and applied voltage (*V*) as,

*P*_*PD*_ = *I*_*leakage*_·*V* (5)

This paper tries to find a simple relationship between the power dissipated by the PDs and the energy of the digital images, which is associated with the corona effect. Therefore, a linear relationship between the leakage current and the applied voltage simplifies the finding of a relationship between the power dissipated by the PDs and the energy of the digital images

**Figure 6:**
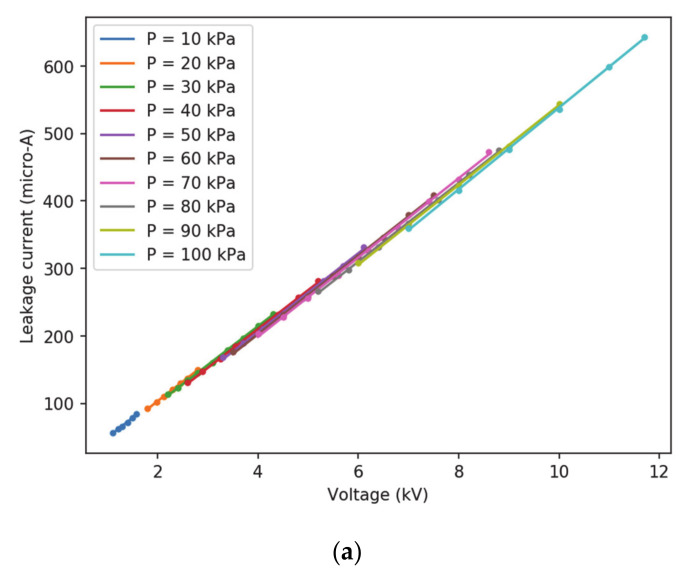

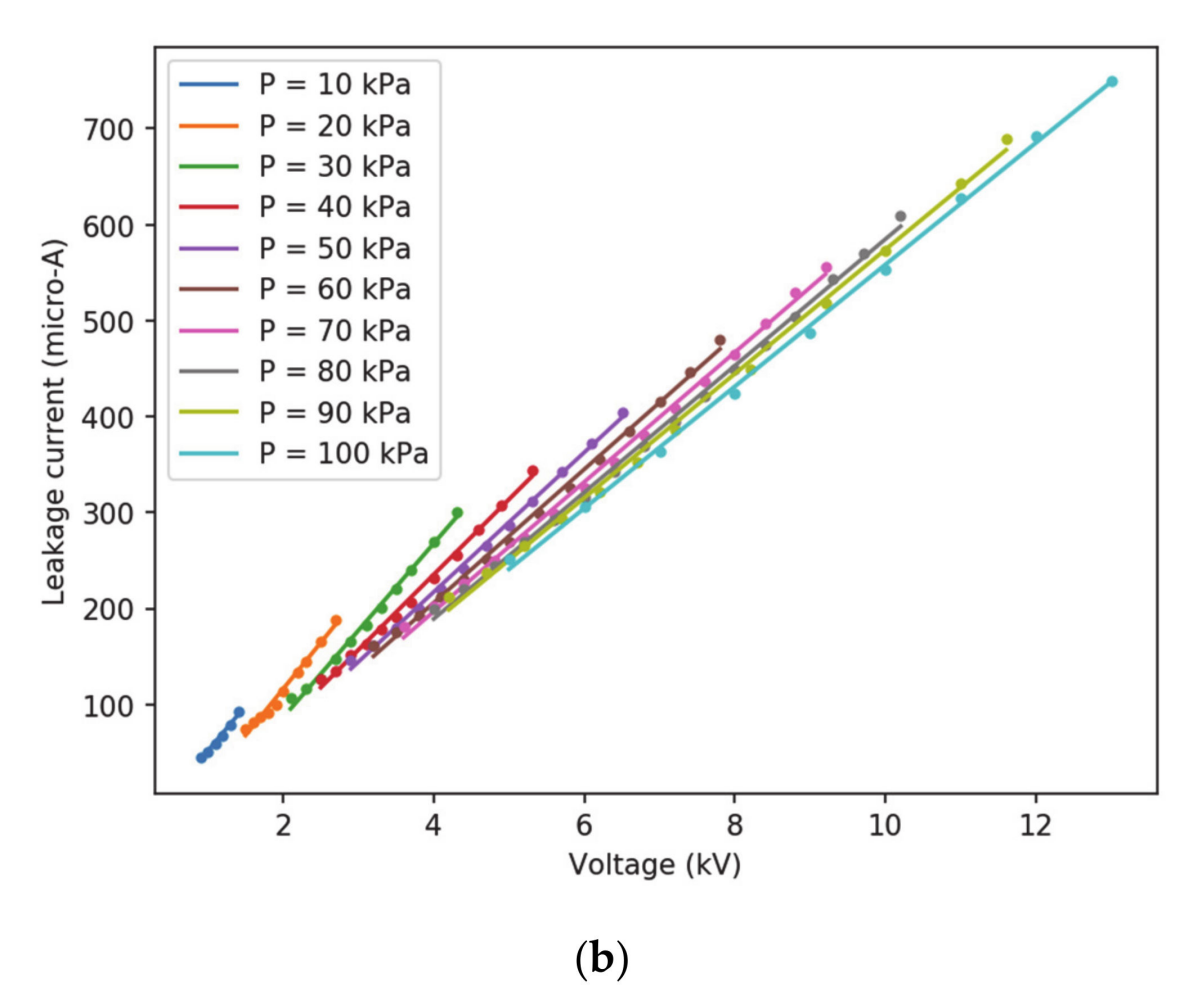
Leakage current versus applied voltage. (**a**) Positive DC supply. (**b**) Negative DC supply.

Figure 6 shows the experimental relationship between the leakage current and the applied voltage under positive DC and negative DC supply, respectively. The results of the linear fitting between the leakage current and the applied voltage are summarized in Table 1. These results show a good linear correlation between the leakage current and the applied voltage.

### 4.3. Relationship between the Energy of the Images and the Electrical Power Dissipated by the PDs

In this subsection the energy of the images is correlated to the electrical power dissipated by the PDs. This is done to determine whether they are interrelated in order to assess the severity of insulation faults from the energy of the digital images acquired by the low-cost imaging sensor.

**Figure 7:**
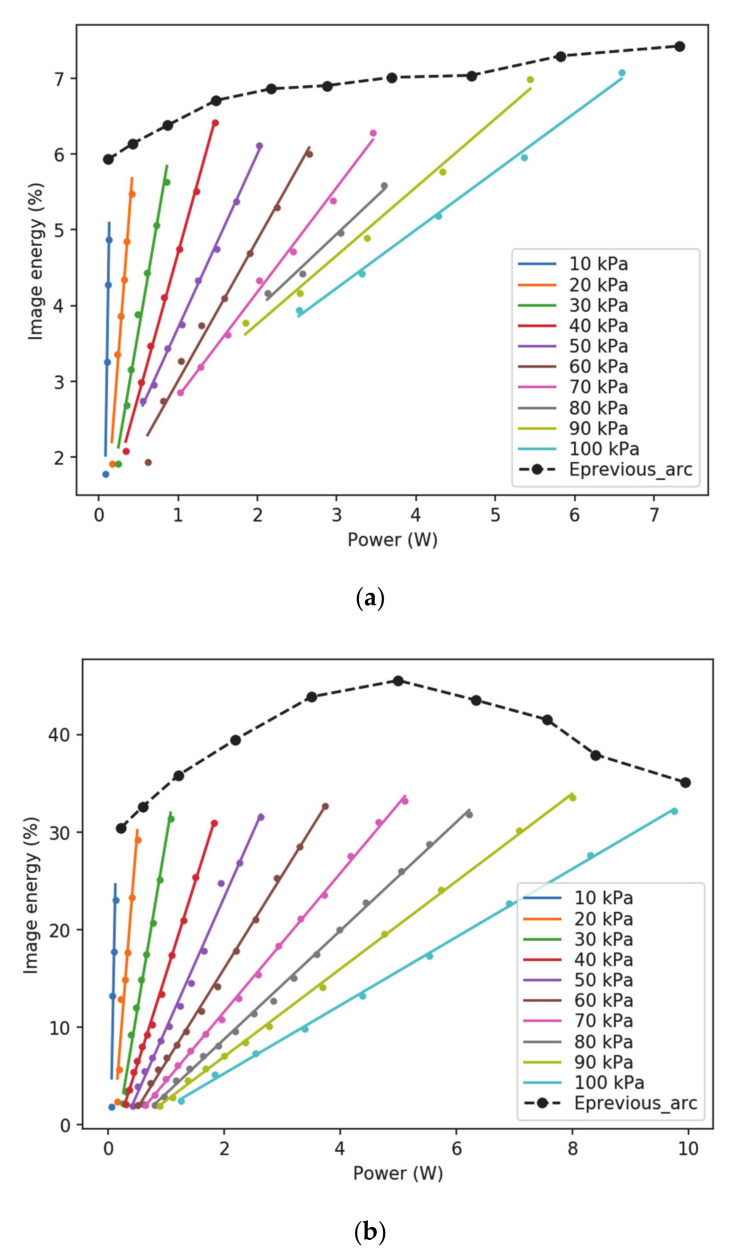
Energy of the images versus the electrical power dissipated by the PDs. The dashed black line (*E_Previous_arc_*) sets the limit energy just before arc occurrence. (**a**) Positive DC supply. (**b**) Negative DC supply.

Figure 7 displays the experimental relationship between the energy of the images once converted to gray level and the electrical power dissipated by the partial discharges, calculated as the product between the leakage current and the applied voltage, i.e., *P_PD_* = *I_leakage_·V*. The results of the linear fitting between the energy of the images and the electrical power of the PDs are summarized in Table 2. These results suggest a good linear correlation between the energy of the images and the electrical power dissipated by the PDs.

Results summarized in Figure 7 and Table 2 show a linear behavior of the energy of the images versus the electrical power dissipated by the partial discharges, for both positive and negative DC supply. Therefore, it can be deduced that it is possible to infer the health status of the insulation from the energy of the corona images. Figure 7 also shows the limit for the arcing condition for each of the considered pressures. This information will be used in the next subsection, where a criterion for classifying the corona severity is proposed.

**Figure 8:**
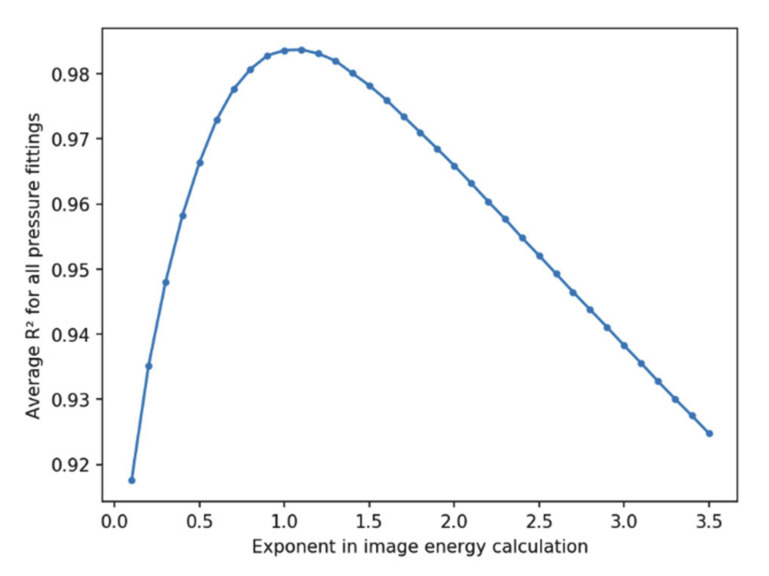
Detail of how the quality of the linear fitting between electrical power and image energy varies as a function of the chosen exponent *k* in (3) for image energy calculation ranging from 0.1 to 3.5.

Given the disagreement found in several references regarding the adequate exponent for image energy computation (1 or 2, mainly) in Equations (3) and (4), it is worth studying its effect on the relationship between the energy of the images and the electrical power dissipated by the PDs. Figure 8 shows the *R*^2^ metric for the linear fittings as a function of the chosen exponent to compute the energy of the images. As can be observed, the value of 2 does not yield a suitable linear relationship, whereas an exponent close to 1 yields the maximum value of the coefficient of determination *R*^2^. Therefore, a value of 1 has been chosen in this work.

### 4.4. Criterion to Determine the Early Appearance of Insulation Faults

Along the presented experiments, it has been proven that the electrical power dissipated by the PDs follows a linear relationship with the image energy captured by a visible UV imaging sensor. This opens a broad new methodology for incipient PD detection, thus allowing predictive maintenance tasks in MEAs based on low-cost imaging sensors to be complimented. It is appealing since imaging sensors are able to localize the fault source without the interference of electrical noise present in an aircraft.

**Figure 9:**
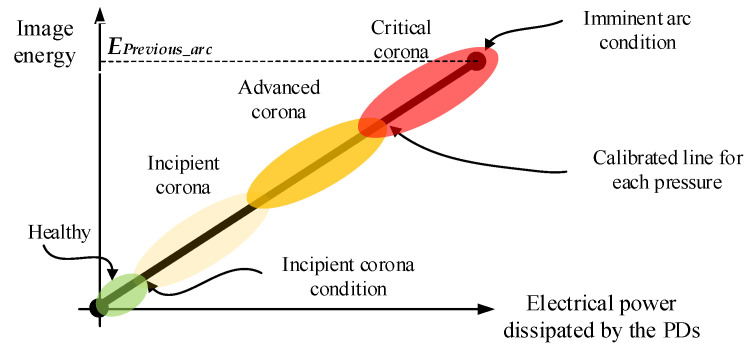
Proposed insulation fault severity chart based on the calibrated line for each particular setup and pressure.

Figure 9 proposes a three-level alert strategy to detect early insulation faults, which is common in other areas [42], i.e., the incipient corona condition, advanced corona and critical corona regions which may led to several actuations or maintenance strategies as soon as a determined level is achieved.

This paper suggests delimiting the four areas as follows:

Healthy condition, between 0% and 10% of *E_Previous_arc_*;
Incipient corona, between 10% and 40% of *E_Previous_arc_*;
Advanced corona, between 40% and 70% of *E_Previous_arc_*;
Critical corona, between 70% and 100% of *E_Previous_arc_*.

Although three equally spaced severity levels have been proposed, the established levels and thresholds may vary depending on the application.

The imminent arc condition for every pressure has been experimentally characterized as shown in Figure 8, indicated by the dashed black line, both for positive and negative DC supplies. As an application example, consider the energy previous to the arc condition of *E_Previous_arc_* = 45.2% for a pressure of 60 kPa and negative DC supply. In this case, the four previously defined zones according to the proposed threshold are bounded by the following image energy levels:

Healthy condition: image energy values between 0 and 4.5%;
Incipient corona: image energy values between 4.5% and 18.1%;
Advanced corona: image energy values between 18.1% and 31.6%;
Critical corona: image energy values between 31.6% and 45.2%.

The continuous image energy monitoring will allow the alarm to be sounded as the corona evolves towards the critical zone and therefore apply the corresponding corrective maintenance measures to avoid further insulation degradation.

## 5. Conclusions

Insulation faults are troublesome since they are difficult to detect in the early stage due the low level of activity. Current electrical protections react when the insulation fault is very advanced because of this low activity level in the early stage and, thus, the fault remains undetected although their continuous activity progressively damages insulation systems until complete failure. In addition, such protections are unable to locate the source of the fault. This paper has proposed a solution to solve these issues, since there is an imperative need to improve current protections. To this end, the optical radiation (visible UV) emitted by the PDs originated during the premature stage of insulation damage is sensed using low-cost, high-resolution imaging sensors and analyzed. This analysis has been carried out by applying simple image processing techniques based on the energy of the digital images. The experimental part has been based on a point-to-plane gap configuration placed inside a low-pressure chamber to simulate the pressure environment of aircraft systems. Digital photographs of the PDs were taken in the 10–100 kPa range, covering pressure range typical of aeronautic applications, and further post processed to determine their energy content. First, it has been proved that the electrical power dissipated by the PDs is proportional to the energy contained in the acquired digital images, which show the visible UV light emitted by the PDs. Therefore, from the energy of the corona images, it is possible to infer the health status of the insulation. A criterion to quantify the severity of insulation faults based on the energy of the corona images, which deals with three equally spaced severity levels, has been proposed. The thresholds for each level may vary depending on the specific configuration dealt with and the criterion of the reliability engineers, thus requiring a prior calibration. The calibration is also required by most commercially available PD sensors, which are often very expensive and do not allow the PD sources to be located. Therefore, the proposed method, apart from allowing the severity of insulation faults to be quantified, also locates accurately the exact location of the insulation region generating PD activity. The accuracy in locating the source of the insulation faults depends on its size, the total area of the image and the number of pixels of the image. The findings of this work allow the fault to be anticipated before it produces irreversible damaging effects, while offering the possibility to complement existing electrical protections, which trip when the arc has developed.

The method proposed in this paper has been tested in a simulated aircraft environment, although it can be applied to many other industrial applications.

## Figures and Tables

**Table 1:** DC supply. Main parameters of the linear regression between the leakage current and the applied voltage (*I_leakage_ = I_o_ + k*_1_*·V*).

	Parameters	10 kPa	20 kPa	30 kPa	40 kPa	50 kPa	60 kPa	70 kPa	80 kPa	90 kPa	100 kPa
**Positive DC**	*I_o_* (μA)	−7.8	−12.7	−14.9	−20.2	−27.4	−31.0	−36.4	−39.2	−49.2	−66.6
	*k*_1_ (μA/kV)	57.5	57.9	57.3	57.6	58.3	58.3	58.7	58.2	59.1	60.5
	*R* ^2^	0.995	0.999	0.999	0.999	0.998	0.999	0.999	0.999	0.999	1.000
**Negative DC**	*I_o_* (μA)	−42.1	−77.3	−95.0	−78.4	−74.0	−72.5	−73.6	−74.8	−73.2	−76.7
	*k*_1_ (μA/kV)	93.7	96.7	90.7	78.4	72.8	69.6	67.6	66.0	64.7	63.5
	*R* ^2^	0.983	0.987	0.994	0.996	0.997	0.997	0.998	0.998	0.998	0.999

**Table 2:** DC supply. Main parameters of the linear regression between the energy of the images and the power dissipated by the PDs (*E_images_ = E_o_ + k*_2_*·P_PD_*).

	Parameters	10 kPa	20 kPa	30 kPa	40 kPa	50 kPa	60 kPa	70 kPa	80 kPa	90 kPa	100 kPa
**Positive DC**	*E_o_* (−)	−3.591	−0.066	0.681	0.919	1.406	0.944	1.414	1.979	1.951	1.770
	*k*_2_ (−/W)	65.41	13.68	5.930	3.773	2.298	2.024	1.382	0.988	0.903	0.810
	*R* ^2^	0.960	0.974	0.989	0.997	0.997	0.981	0.995	0.984	0.991	0.995
**Negative DC**	*E_o_* (−)	−15.44	−7.399	−5.027	−3.421	−3.664	−3.196	−2.671	−2.410	−2.053	−1.786
	*k*_2_ (−/W)	311.0	74.00	33.08	18.83	13.46	9.581	7.106	5.571	4.499	3.501
	*R* ^2^	0.904	0.962	0.986	0.998	0.992	0.998	0.999	0.999	0.999	0.999
